# Identification, characterization and functional analysis of grape (*Vitis vinifera* L.) mitochondrial transcription termination factor (mTERF) genes in responding to biotic stress and exogenous phytohormone

**DOI:** 10.1186/s12864-021-07446-z

**Published:** 2021-02-26

**Authors:** Xiangjing Yin, Yu Gao, Shiren Song, Danial Hassani, Jiang Lu

**Affiliations:** 1 Center for Viticulture and Enology, School of Agriculture and Biology, Shanghai Jiao Tong University, Shanghai, 200240 China; 2 Joint International Research Laboratory of Metabolic and Developmental Sciences, Key Laboratory of Urban Agriculture (South) Ministry of Agriculture, Plant Biotechnology Research Center, Fudan–SJTU–Nottingham Plant Biotechnology R&D Center, School of Agriculture and Biology, Shanghai Jiao Tong University (SJTU), Shanghai, 200240 China

**Keywords:** Bioinformatics analysis, Expression profile analysis, Grapevine (*V. vinifera* L.), mTERF family

## Abstract

**Background:**

Mitochondrial transcription termination factor (mTERF) is a large gene family which plays a significant role during plant growth under various environmental stresses. However, knowledge of mTERF genes in grapevine (*Vitis* L.) is limited.

**Results:**

In this research, a comprehensive analysis of grape mTERF (*VvmTERF*) genes, including chromosome locations, phylogeny, protein motifs, gene structures, gene duplications, synteny analysis and expression profiles, was conducted. As a result, a total of 25 mTERF genes were identified from the grape genome, which are distributed on 13 chromosomes with diverse densities and segmental duplication events. The grape mTERF gene family is classified into nine clades based on phylogenetic analysis and structural characteristics. These *VvmTERF* genes showed differential expression patterns in response to multiple phytohormone treatments and biotic stresses, including treatments with abscisic acid and methyl jasmonate, and inoculation of *Plasmopara viticola* and *Erysiphe necator*.

**Conclusions:**

These research findings, as the first of its kind in grapevine, will provide useful information for future development of new stress tolerant grape cultivars through genetic manipulation of *VvmTERF* genes.

**Supplementary Information:**

The online version contains supplementary material available at 10.1186/s12864-021-07446-z.

## Background

In eukaryotes, genetic information is not only stored in the nucleus, but also in organelle genomes such as mitochondria and chloroplasts. However, these organelles’ gene pool has dramatically reduced during their evolution, which is due to the loss of their genes, and continuous transfer of organelle-nuclear genes [1–3]. In living organisms, the organelle gene expression system largely depends on nuclear-coding proteins, which include RNA polymerase, sigma factor, as well as specific RNA maturation factors [4–8]. Meanwhile, some organelle protein families including, PPRs, HAT, OPRs and mTERFs which have similar modular structures consisting of repetitive helical motifs also play an important role in their gene expression mechanism [4, 9].

Mitochondrial transcription termination factor (mTERF) genes comprise a large family which plays an essential role in the regulation of mitochondrial gene transcription [10]. MTERF proteins possess a unique repetition of 30 amino acids residue, which enables them to recognize and bind to specific sites on mitochondrial genome known as typical mTERF motif [11]. In human for instance, mTERFs comprises a proline at position 8, 11, 18 and 25. Therefore, the motif is conserved for leucine or hydrophobic amino acids, indicating that there are at least three leucine motifs in the mTERFs [12]. Former research indicated that mTERF proteins could have multiple biological functions of intracellular regulation. For instance, human mTERF1, with 342 amino acids in length, can bind to 28 nucleotide sequences downstream of the 3’end of 16SrRNA, leading to the termination of mitochondrial gene transcription [13, 14]. The mTERF1 protein possess regulatory function for transcriptional initiation of mitochondrial rDNA and mitochondrial DNA replication [15]. In addition, mTERF2 protein showed a significant downregulation of mitochondrial transcription level in vitro, suggesting that mTERF2 protein may affect mitochondrial transcription by binding with regulatory activators of mtDNA transcription initiation [16].

In recent years, plant mTERF genes and their roles in mitochondrial gene expression regulation have received a good deal of attention [17]. Bioinformatic analysis shows that these mTERF genes are a large and complex family existing in metazoans and plants [18]. There are at least identified 35 mTERFs in *Arabidopsis thaliana*, mainly located in mitochondria or chloroplasts, participating in abiotic stresses [19, 20]. For instance, the seed germination rate of *mterf1* (*soldat 10*) mutant was considerably lower than wild type under the same condition [21]. Over expression of the *AtmTERF5* (*MDA1*) gene affects the germination rate of transgenic lines under simulated drought stress as higher germination rate was observed under mannitol treatment [22]. Besides, *A. thaliana* mterf9 mutant was insensitive to ABA treatment. Under the treatment of NaCl and ABA, the root growth retardation of mTERF9 mutants displayed the phenotype of short root and lighter fresh weight compared to the wild type [23]. Furthermore, previous studies in maize showed that ZmmTERF4 protein can co-immunoprecipitated with multiple chloroplast introns leading to the disruption of splicing in Zm-mterf4 mutants, indicating its key role in meditating the communication between organelle and the nucleus [24]. The evidence expands the functional knowledge of the mTERF family.

As a large economic worth fruit crop [25], grapevine is an important candidate for identifying stress resistance genes to leading to better grape quality. At present, the basic structure and preliminary functions of mTERF family proteins have been continuously explored, but their detailed functions and regulation mechanisms under different stresses still remain unknown. This study introduces the members of the grape mTERF gene family (*VvmTERF*) and determine their potentiality in stress resistance, aiming to afford an essential information of the grape mTERF gene family and providing a resource for functional research in grape biology study.

## Results

### Identification of *mTERF* genes in grape genome

MTERF genes in the grape genome were identified by BLASTP with HMMER 3.0 [26] searching key domain mTERF PFAM file (PF02636) and previous reports [20, 27]. A total of 25 grape mTERF genes were identified, which were named as *VvmTERF1*-*VvmTERF25* according to sequence of their chromosomal locations (Table 1). A high conserved mTERF domain was found in all the VvmTERF proteins.

**Table 1:** The grape mTERF gene family

Protein name	Gene locus	Chromosome location	Strand	CDS (bp)	Protein (aa)	mTERF domain location (aa)	E-value
VvmTERF1	GSVIVT01010499001	chr1: 21096155...21107668	*–*	822	273	99–237	5.32e-24
VvmTERF2	GSVIVT01023845001	chr3: 3030639...3032099	*–*	1065	354	72–344	1.16E-54
VvmTERF3	GSVIVT01031956001	chr3: 5686087...5687004	*–*	831	276	26–271	1.39e-31
VvmTERF4	GSVIVT01031970001	chr3: 5802834...5807050	*–*	1581	526	149–455	2.34E-70
VvmTERF5	GSVIVT01017772001	chr5: 3341975...3348425	*–*	1284	427	49–165	5.59E-17
						213–358	8.20E-05
VvmTERF6	GSVIVT01011061001	chr7: 1887643...1890890	+	1596	531	55–490	0.00E+ 00
VvmTERF7	GSVIVT01010970001	chr7: 2517645...2525482	+	1227	408	84–357	1.88E-33
VvmTERF8	GSVIVT01028380001	chr7: 6844159...6845397	+	1239	412	97–371	7.82E-41
VvmTERF9	GSVIVT01028382001	chr7: 6850176...6869742	+	2367	788	120–339	1.64E-30
						409–738	5.17E-41
VvmTERF10	GSVIVT01028383001	chr7: 6873625...6888273	+	2658	885	107–381	4.50E-23
						488–803	2.62E-35
VvmTERF11	GSVIVT01028384001	chr7: 6891622...6892722	+	1101	366	61–315	1.29E-27
VvmTERF12	GSVIVT01022213001	chr7: 17541013...17544022	+	1110	369	66–341	6.11E-32
VvmTERF13	GSVIVT01033517001	chr8: 20068603...20072280	*–*	1770	589	280–564	6.81E-16
VvmTERF14	GSVIVT01029533001	chr9: 21885971...21897453	*–*	2427	808	267–574	6.49E-130
VvmTERF15	GSVIVT01021544001	chr10: 6867305...6869519	+	738	245	139–227	9.50E-20
VvmTERF16	GSVIVT01026275001	chr10: 15271383...15274580	*–*	1692	563	254–520	1.91E-10
VvmTERF17	GSVIVT01015207001	chr11: 1833849...1837293	*–*	1662	553	17–338	0.00E+ 00
VvmTERF18	GSVIVT01012810001	chr11: 5607921...5618868		2160	719	486–637	1.50E-12
VvmTERF19	GSVIVT01001819001	chr14: 26071265 ...26073597	+	1395	464	192–449	4.80E-49
VvmTERF20	GSVIVT01038641001	chr16: 21269851...21283495	*–*	5655	1884	196–492	4.90E-32
VvmTERF21	GSVIVT01008120001	chr17: 5628041...5629396	*–*	1356	451	84–278	4.88E-10
VvmTERF22	GSVIVT01009012001	chr18: 4269303...4275210	*–*	1278	425	86–353	2.86E-26
VvmTERF23	GSVIVT01034475001	chr18: 20728900...20735286	*–*	639	212	125–196	1.86E-08
VvmTERF24	GSVIVT01037780001	chr19: 7803504...7814106	+	1443	480	195–470	1.09E-40
VvmTERF25	GSVIVT01036787001	chr19: 22546264...22547496	+	1233	410	94–368	3.68E-45

### Phylogenetic analysis and classification of grape *mTERF* genes

**Fig. 1:**
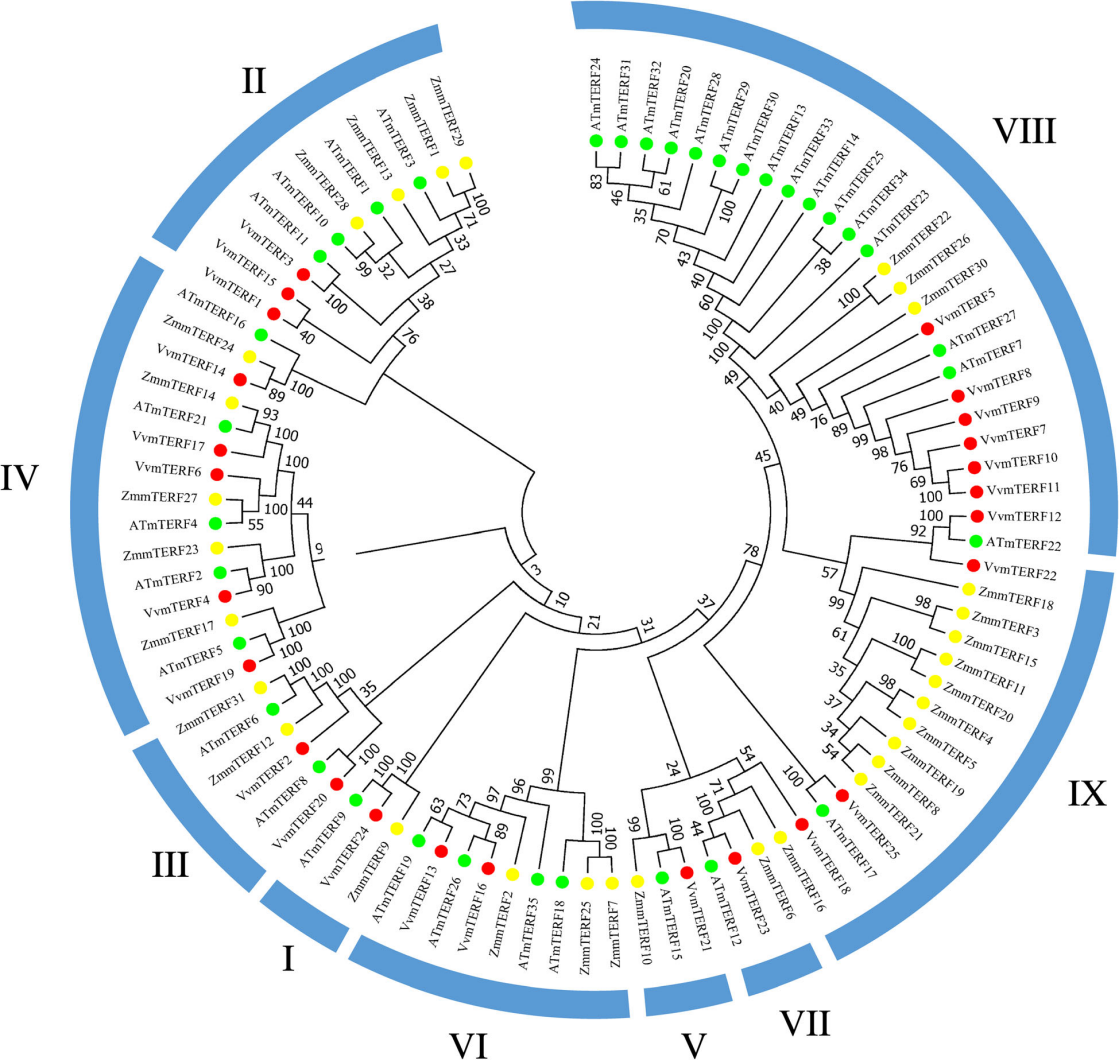
Phylogenetic analysis among the grape, Arabidopsis and maize mTERF proteins. The unrooted tree was constructed using MEGA5.0 software by Neighbor-joining method. The numbers represent the bootstrap values (%) for 1000 bootstrap replicates and only bootstrap values > 60% are shown. Nine groups designated I–IX are shown outside. Three dot colors mean different species. Yellow, green and red represent maize, Arabidopsis and grape, respectively

In order to evaluate the evolutionary relationship of *VvmTERF* gene family, a total of 91 mTERF genes from Arabidopsis (35), maize (31) and grape (25) genomes were collected for a phylogenetic tree construction using MEGA5.0 software (Fig. 1 and Figure S1). Detailed sequence information of Arabidopsis and maize mTERF genes were obtained from a previous study [28]. The tree topology result demonstrated that nine groups (Clade I–IX) were classified according to homologous genes of maize and Arabidopsis. Of the 25 *VvmTERF* genes, Clade VII contained 7 genes, the most among all the clades, while other clades had 1 to 5 members, respectively. One grape mTERF gene, *VvmTERF24*, belonged to Clade I where 2 members were identified in Arabidopsis and in maize [20, 28]. It is worth noting that the well functional characterized mTERF genes from Arabidopsis, such as *SOLDAT10* (*AtmTERF1*, AT2G03050), *BSM/RUG2* (*AtmTERF4*, AT4G02990), and *SHOT1* (*AtmTERF18*, AT3G60400) were distributed in group II, IV and VI, respectively. Meanwhile, a certain of grape mTERF genes belong to these groups, indicated their close evolutionary relationships with Arabidopsis mTERF genes from the same group.

### Exon–intron structure analysis of *VvmTERF* genes

**Fig. 2:**
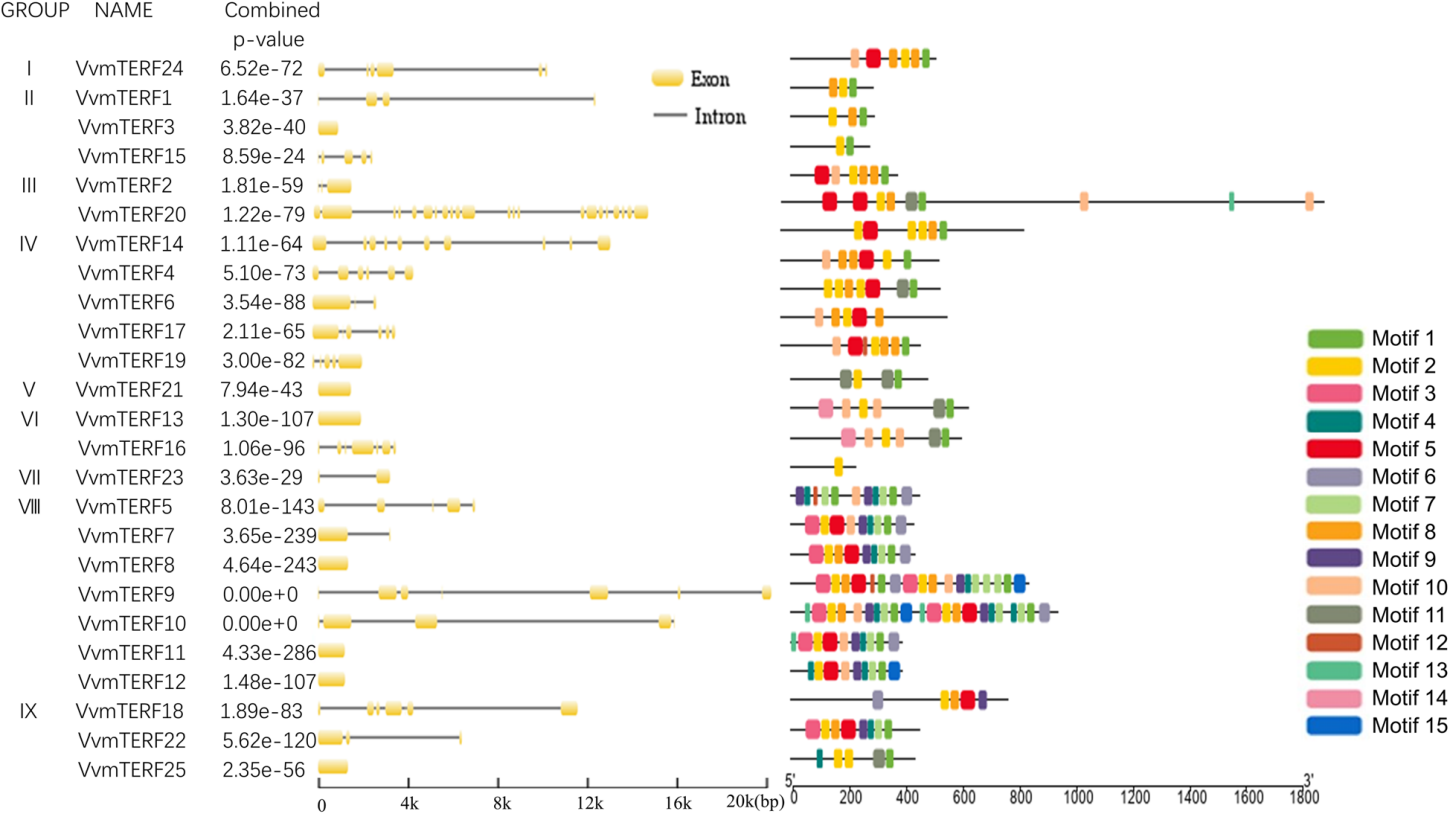
**a** Sequence analysis of introns and exons in grape mTERF genes. The yellow boxes and dark lines represent exons and introns sequences, respectively. **b** Schematic diagram of predicted recognized conservative modules in grape mTERF protein. The MEME program was used to mine the presumptive conservative motif of grape mTERF protein. Different colored boxes were used to show putative fifteen motifs and the sequences of regular motifs were displayed in the Table 2

Structure analysis on the exon and intron boundaries of the *VvmTERF* genes will provide important clues as they played significant roles in evolution of various gene families. The number of exons per grape mTERF gene ranged from 1 to 22 (Fig. 2). Among them, *VvmTERF20* had the highest number of exons of 22, followed by *VvmTERF14* (10), *VvmTERF16* (7), *VvmTERF18* (6), *VvmTERF*9 (6), *VvmTERF24* (6) and *VvmTERF4* (6), while *VvmTERF3*, *VvmTERF8*, *VvmTERF11–13* and *VvmTERF21* had only one exon each. These results indicated that during the long evolution of *VvmTERF* gene family, both exon loss and gain have occurred, which might lead to diversified function among the other closely related mTERF genes. In clade IV, for instance, the number of exons was quite large, ranging from three to ten, while the genes in clade I and IX had a relatively smaller number, ranging from one to six exons. It is interesting that *VvmTERF7*, *8*, *11* and *12* demonstrated similar exon/intron structures and came from the same clade while most VvmTERF genes showed distinct structures. This difference in exon/intron patterns might be resulted from a series of gene replication events.

### Conserved motifs and subcellular localization analysis of *VvmTERF*

Searching for putative conserved motifs in grape mTERF proteins analysis was conducted via Pfam [29] and SMART [30] databases. In order to predict the potential motifs in the putative grape mTERF gene family gene sequences, the MEME (Multiple Em for Motif Elicitation) program [31] was used and 15 mTERF motifs in grape were identified and clustered (Fig. 2 and Table 2) using the ClustalW 2.0 program [32]. Among all, class VII sequences had more than 10 mTERF motifs, and clade IX mTERF sequences showed 5–8 mTERF motifs. Identified in human mTERF proteins previously [12], conversed mTERF motifs containing repeats of leucine zipper-like heptad X_3_LX_3_ structure was also found in grape mTERF motifs (Table 2), suggesting that fundamental structures and functions of mTERF proteins in *Vitis* might be similar to human mTERF proteins.

**Table 2:** Multiple Em for Motif Elucidation (MEME) protein motifs identified in grape mTERF proteins

Motif No.	Width	Sequence
1	27	ELVRFPQYLSYSLEKRIKPRHSVVKV
2	30	KIVTKYPEJLGASVEKTLKPKLEYLKSLG
3	51	HSFTVSYLMNSCGLSPETAISASKKIQFENPENPDSVLALLRNHGCTDTH
4	24	ESTWZQKMEVYRRWGFSEDEI
5	51	SDSDVAKIJKKRPRILKYDLEKNJKPNIEFLKEJGIPDSSIAKVIARYPR
6	38	AFLKLTEKKFLDRFVIKYZEDVPQLLNLYKGEVGIQE
7	26	AFRKSPLCMQLSEKKIMSTMDFLVN
8	30	DIARILSKYPQILGRSJENNLKPSVNYLV
9	30	ENVKKVMEMGFBPLKLTFVYAJQVISQMS
10	31	EENJLPNJAYLEEJGVPRSQISKLLTRYP
11	42	KYGLSEEEVSEMFKKAPQVLQYSEDKIEEKIDYLVNKMGYP
12	16	CMSLSEKKIMSTMDF
13	21	MTQLHFLGNITPFVIRCF
14	51	HCTRSFQFMDAENMSKNSPFFLZKJLGKVENEQEIGKSJSKFLRYNPINE
15	41	KKDLKLGHFLNLPEGDFLDKYVIKNQDEIPQLLDLYQGKV

Aiming to find predicted motifs shared among related proteins within the grape mTERF gene family, the MEME database program [31] was performed. As shown in Fig. 2, a total of 15 motifs were discovered in these 25 proteins. Among them, motifs 2 and 8 were found in most grape mTERF proteins. Motif sequences comparison with PFAM mTERF domain alignment revealed that motifs 1, 4 and 5 partly covered the PFAM mTERF domain (PF02536), and motif 5 belonged to specific organelle-targeting mTERF proteins, such as the group IV grape mTERF proteins (Fig. 2). It is highly probabe that group-specific motifs lead to characteristic functions in various life activities.

In plants, the subcellular localization of a protein is closely related to its biological function. Table 3 indicates the predicted cellular location of VvmTERF proteins for future functional research. Based on protein sequence, subcellular localization prediction demonstrated that there are 12 VvmTERFs associated with chloroplasts or mitochondria, which may imply that functions of VvmTERF proteins are related to these organelles.

**Table 3:** Subcellular localization of VvmTERF proteins

Protein name	Prediction scores				
	Chloroplast	Mitochondrial	Cytoplasmic	Nuclear	Plasma Membrane
VvmTERF1	0.144	2.201 ^a^	0.211	1.870 ^a^	0.041
VvmTERF2	0.230	1.288 ^a^	1.451 ^a^	1.437 ^a^	0.280
VvmTERF3	0.081	1.936 ^a^	0.162	1.297 ^a^	0.393
VvmTERF4	0.633	0.447	1.125	0.523	2.135 ^a^
VvmTERF5	0.317	0.915	1.094	0.246	1.968 ^a^
VvmTERF6	1.286^a^	0.786	1.684 ^a^	0.567	0.322
VvmTERF7	0.353	1.111	0.177	0.228	2.402 ^a^
VvmTERF8	0.821	1.264 ^a^	0.105	0.445	1.939 ^a^
VvmTERF9	0.333	0.909	0.563	1.033 ^a^	1.551 ^a^
VvmTERF10	0.241	0.789	0.313	0.425	2.649 ^a^
VvmTERF11	0.436	1.159	0.132	0.247	2.339 ^a^
VvmTERF12	0.441	1.388 ^a^	0.296	0.660	1.427 ^a^
VvmTERF13	0.499	1.393 ^a^	1.068 ^a^	0.487	1.193 ^a^
VvmTERF14	0.235	0.738	0.637	2.016 ^a^	1.048
VvmTERF15	0.127	1.210 ^a^	0.404	1.767 ^a^	0.250
VvmTERF16	0.242	0.418	0.343	0.189	3.042 ^a^
VvmTERF17	0.071	0.194	0.279	0.131	4.004 ^a^
VvmTERF18	0.096	0.920	0.607	1.628 ^a^	0.943
VvmTERF19	1.663 ^a^	1.209 ^a^	1.057 ^a^	0.332	0.301
VvmTERF20	0.696	0.283	0.301	0.950	2.268 ^a^
VvmTERF21	0.497	1.564 ^a^	1.782 ^a^	0.556	0.271
VvmTERF22	0.113	3.074 ^a^	0.149	0.963	0.455
VvmTERF23	0.406	0.226	0.891	0.612	0.000
VvmTERF24	0.045	0.342	0.125	3.855 ^a^	0.452
VvmTERF25	0.684	2.162 ^a^	0.205	0.181	1.546 ^a^

The subcellular localization is predicted based on the prediction scores for chloroplast, mitochondria, cytoplasmic, nuclear and plasma membrane location and numbers show the strength of prediction, with large value indicating strong prection

^a^ indicating strong reliability of location

### Synteny analysis of *VvmTERF* and *AtmTERF* genes

**Fig. 3:**
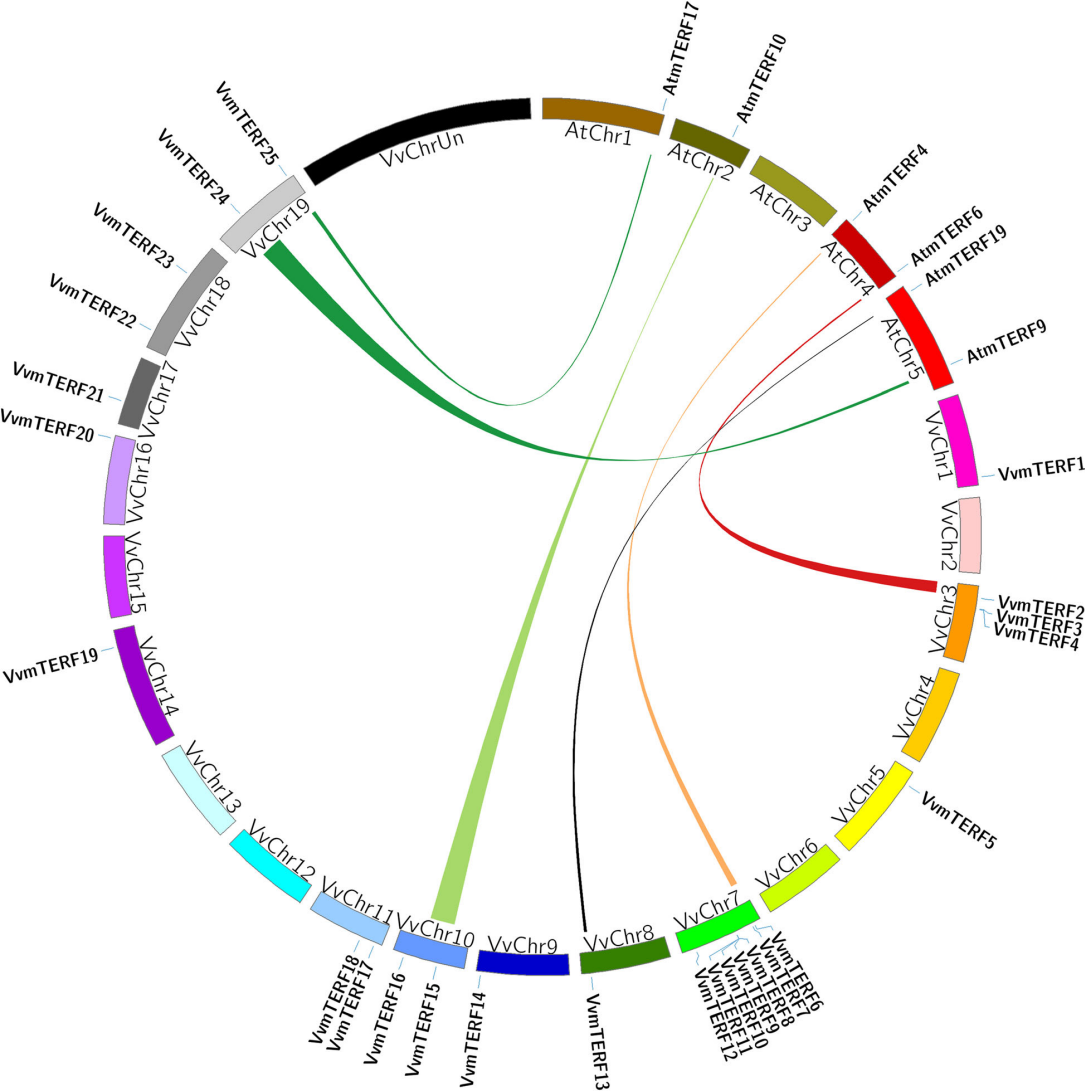
Localization, duplication and synteny analysis of grape mTERF genes. Chromosomes 1–19 are marked using different colors and labeled with their names in a circular form. Syntenic regions are demonstrated by coloured curves between grape and Arabidopsis mTERF genes. Sequence contigs which cannot be located on corresponding chromosomes (1–19) will be assembled on “ChrUn”

Arabidopsis is a well-studied model species which can provide available genomic information to a less-studied species through genomic comparison method [33, 34]. As showed in Fig. 3, a large-scale syntenies study containing 6 pairs of grape and Arabidopsis mTERF genes were recognized. Grape orthologues including *VvmTERF2*, *VvmTERF6*, *VvmTERF13*, *VvmTERF15*, *VvmTERF24* and *VvmTERF25* displayed synteny location with Arabidopsis mTERF genes *AtmTERF6*, *AtmTERF4*, *AtmTERF19*, *AtmTERF10*, *AtmTERF9* and *AtmTERF17*, respectively (Table S2). The number of synteny results indicated that several mTERF genes might arise before the divergence of Arabidopsis and grape lineages, and also suggested that partial deletion of the grape genes might occur in specific syntenic locations during genome evolution.

### Cis-element analysis of grape *mTERF* gene promoters

**Fig. 4:**
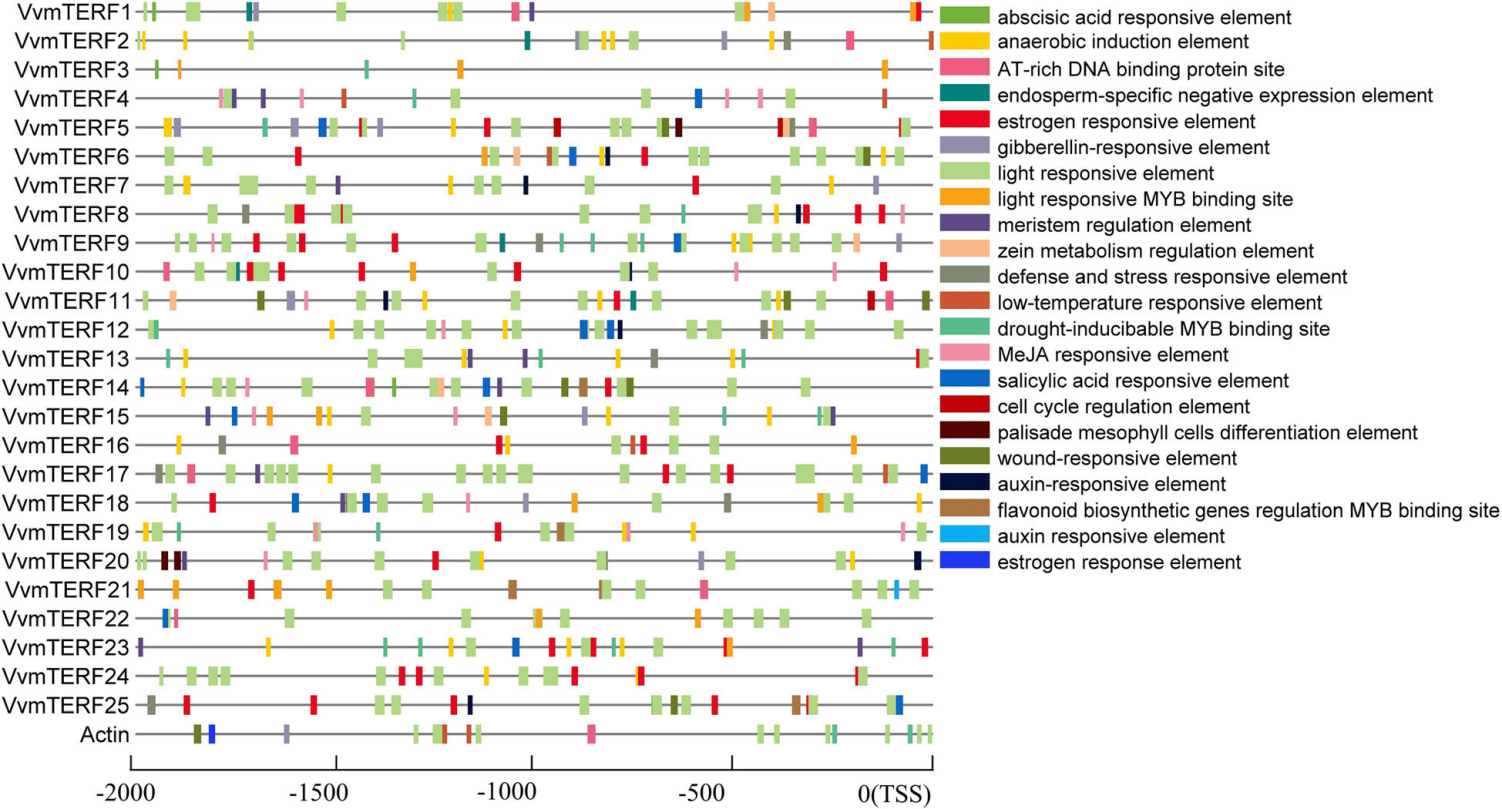
Predicted cis elements of *VvmTERF* gene promoters. Promoter sequences of each *VvmTERF* genes and the housekeeping gene *Actin1* (Accession number AY680701) were analyzed by PlantCARE website (http://bioinformatics.psb.ugent.be/webtools/plantcare/html/)

To understand the possible regulatory mechanism of *VvmTERF* genes in multiple stress responses and functions in chloroplast and mitochondrion, a 2-kb sequence upstream of the precited transcription start site (TSS) of each *VvmTERF* gene was analyzed by the PlantCARE database. Meanwhile, *Actin1* was chosen in grape genome as the housekeeping gene (Fig. 4). The sequences of *VvmTERF* gene promoters were found to contain various hormone regulation-related cis-elements such as those responsive to auxin, MeJA (Methyl Jasmonate), gibberellin, abscisic acid and salicylic acid. In addition, various defense and stress-related elements were also observed. These elements included light and wound responsive elements, osmotic stress-related elements, and low temperature and drought responsive elements.

### Analysis of expression profiles among the grape *mTERF* genes in different tissues and organs

**Fig. 5:**
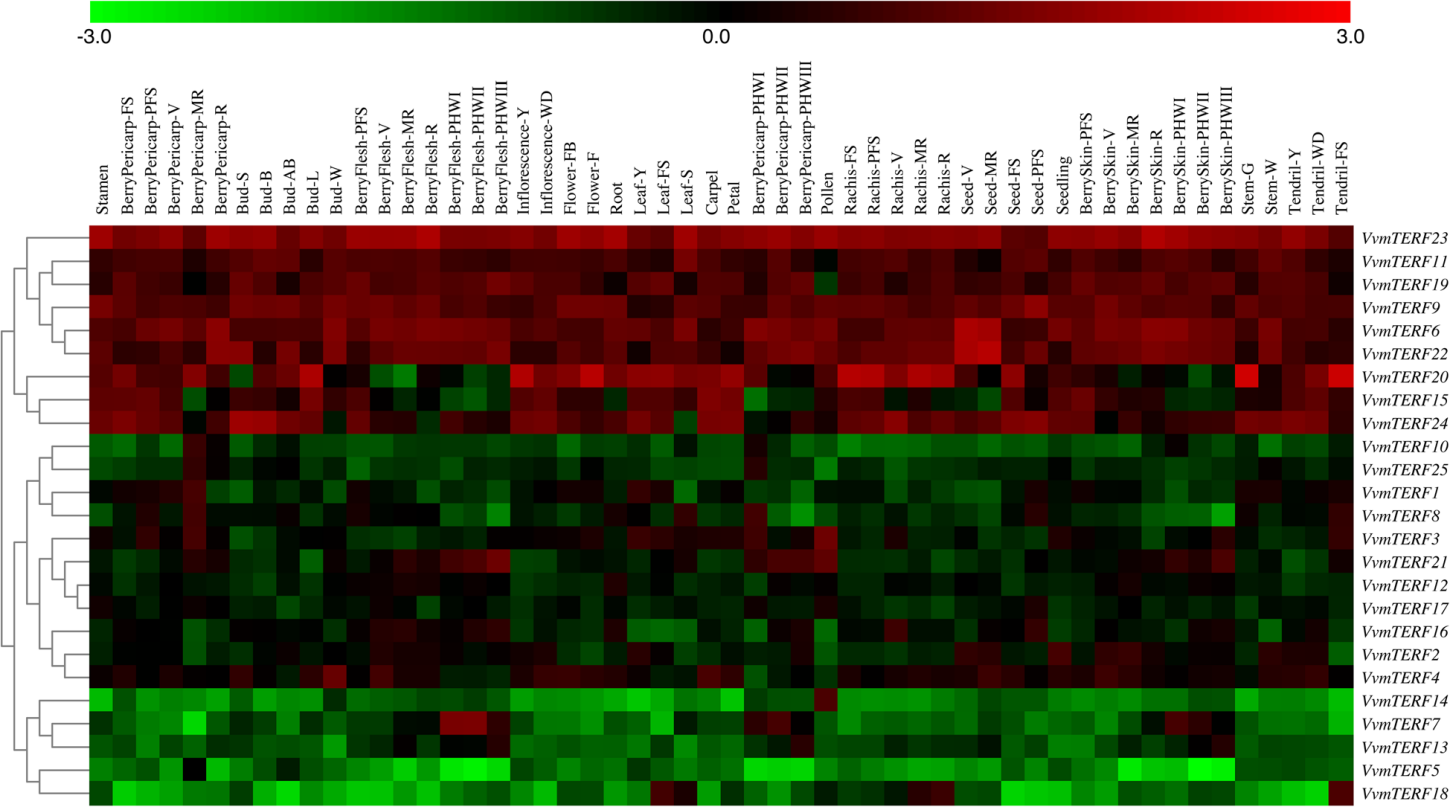
Expression profiles of *VvmTERF* genes in different tissues/organs under various developmental stages. Red and green boxes represent high and low expression levels, respectively. Bud-AB, bud after burst; Bud-B, Bud burst; Bud-W, winter bud; Bud-L, latent bud; Bud-S, bud swell; Flower-F, flowering; Flower-FB, flowering begins; FS, fruit set; Inflorescence-Y, young inflorescence with single flowers separated; Inflorescence-WD, well-developed inflorescence; Leaf-FS, mature leaf; Leaf-S, senescing leaf; Leaf-Y, young leaf; MR, mid-ripening; R, ripening; PFS, post fruit set; Stem-G, green stem; Stem-W, woody stem; V, véraison

To discover the potential function of VvmTERF proteins during different stages of grape development, the tissue/organ-specific gene expression profiles of *VvmTERF* were analyzed in the *V. vinifera* cv. Corvina global gene expression atlas from the GEO DataSet (GSE36128). This dataset contained expression information of 54 sample tissues and organs in different developmental stages acquired by microarray database (Fig. 5). The results showed that some *VvmTERF* genes such as *VvmTERF6*, *9*, *11* and *23* displayed similar expression patterns in different tissues and organs, while other *VvmTERF* genes like *VvmTERF1*, *3*, *10* and *16* demonstrated tissue/organ-specific expression patterns, suggesting multiple roles played by these *VvmTERF* genes family in grapevine.

### Expression patterns of *VvmTERF* genes under different exogenous hormone treatments

**Fig. 6:**
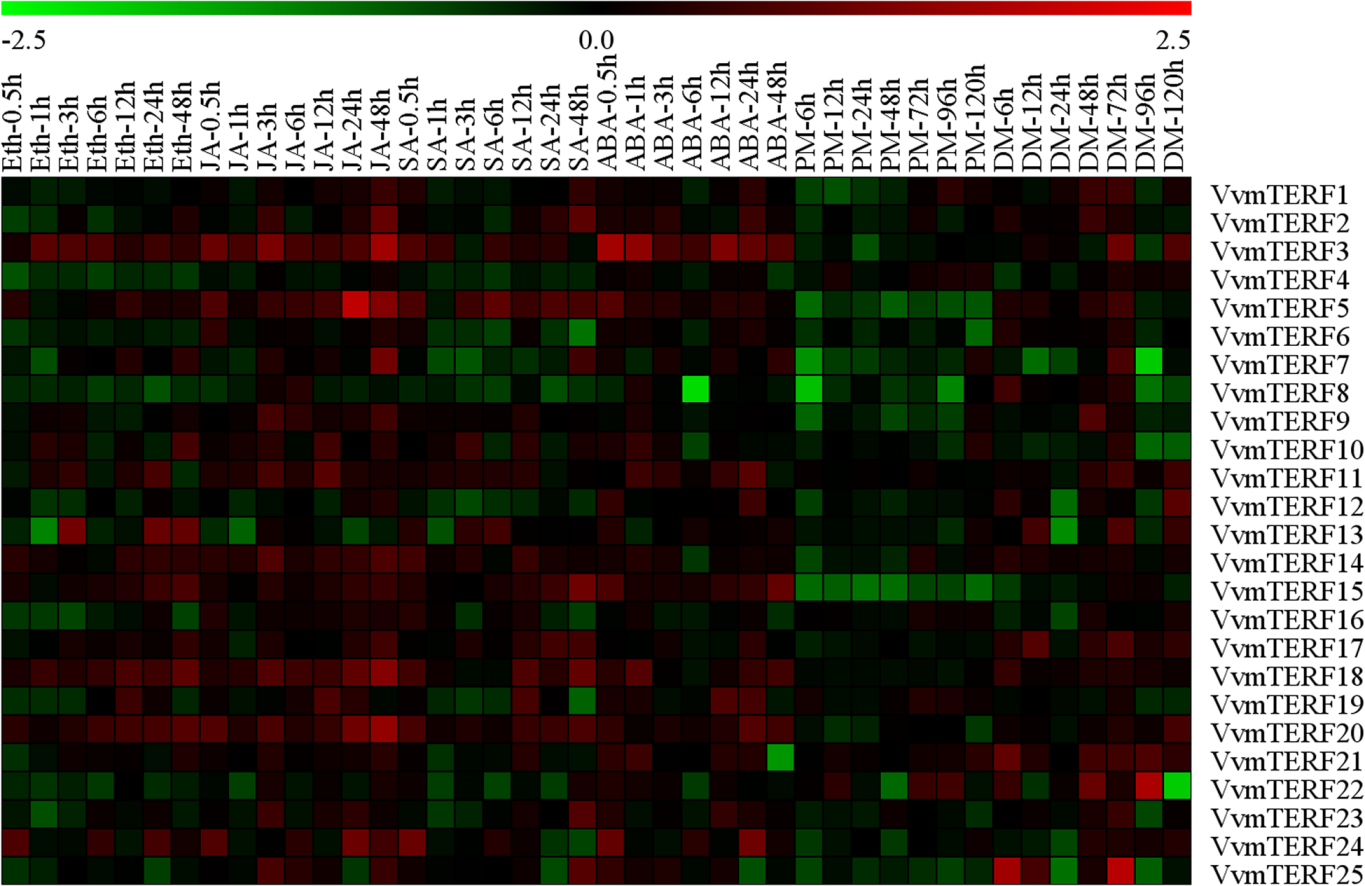
Expression patterns of 25 mTERF genes in grape under treatments of phytohormones and biotic stresses. The Gene Tools software was used to quantify the brightness of semi-quantitative RT-PCR bands. MeV software was used for hierarchical cluster analysis to compare the grape mTERE genes under different hormone treatment and stresses. Red/green were used to indicate increased or decreased expression levels, respectively

To explore potential stress-related genes characterized in this research, plant signaling and regulatory hormones including, ABA, MeJA, SA and Eth were used for exogenous treatment [35]. Interestingly, almost all these *VvmTERF* gene expressions were influenced by exogenous hormone treatments (Fig. 6 and Figure S2). For instance, after the ABA treatment, a total of 13 *VvmTERF* genes displayed multiple degrees of up regulation while 8 genes were down regulated. MeJA treatment led to the expression increase of 17 *VvmTERF* genes and decrease on 7 genes. However, the expression patterns under SA and Eth treatments were different from those regulated by ABA and MeJA as more down regulated genes were observed. A total of 5 *Vv*mTERF genes were up regulated and 12 were down regulated by SA, while 7 were up regulated and 14 were down regulated by exogenous Eth hormone treatment. According to the semi-quantitative RT-PCR result, *VvmTERF2*, *VvmTERF6*, *VvmTERF16*, *VvmTERF22* and *VvmTERF23*, which were downregulated by the Eth treatment, displayed significant upregulation under MeJA treatment, indicating an existence of different regulatory networks among these phytohormones. Meanwhile, the existence number of responsive cis-elements of the promoter region may also play a role in the gene expression regulation.

### Expression profiles of *VvmTERF* genes in response to biotic infections

**Fig. 7:**
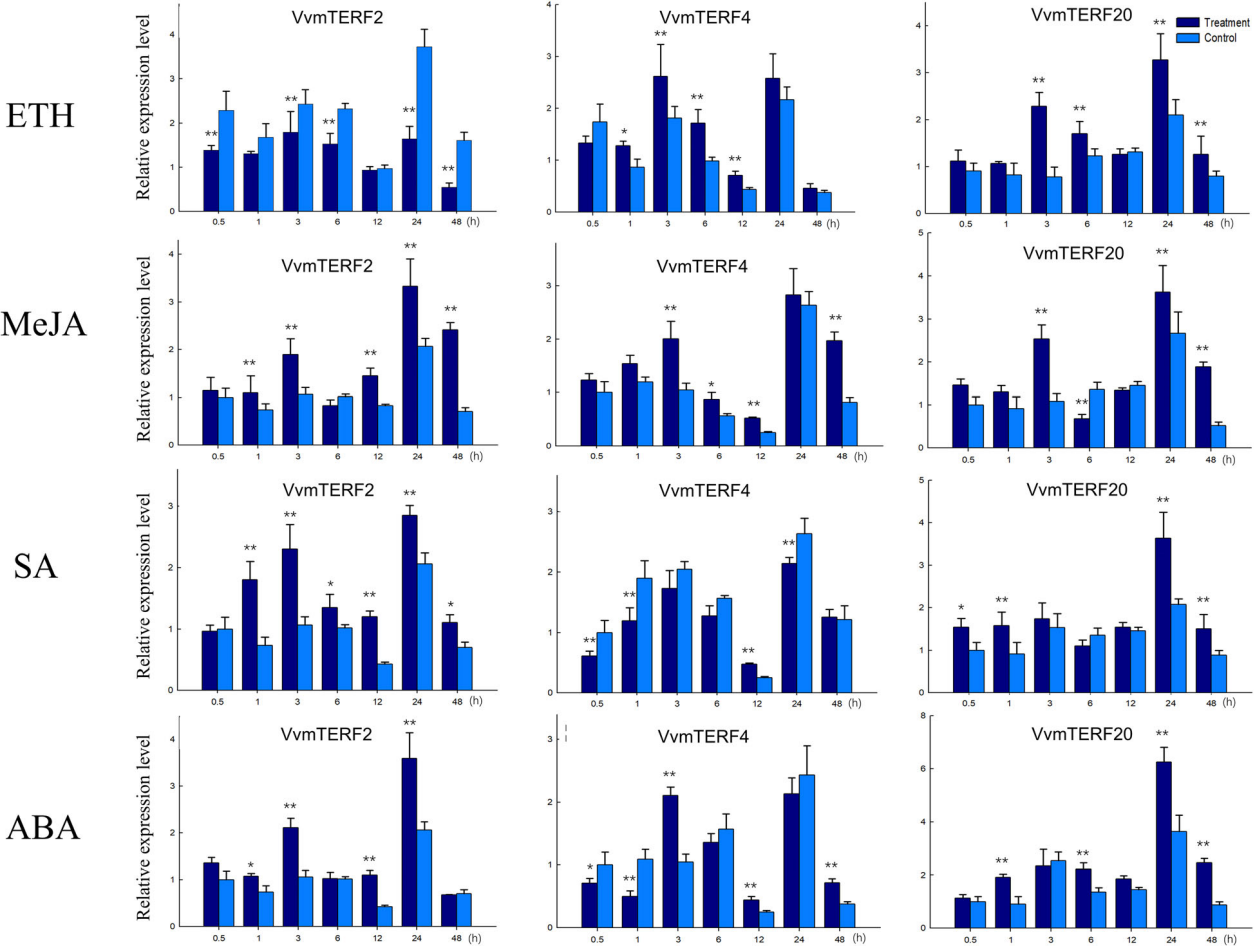
Real-time quantitative PCR expression patterns of 3 selected grape mTERF genes regulated by different hormone treatments including abscisic acid (ABA), salicylic acid (SA), methyl jasmonic acid (MeJA), and ethylene (Eth). Asterisks indicate that the corresponding genes were distinctly up- or down-regulated following various treatments by t-test (*P < 0.05, **P < 0.01). The mean and SD values were derived from three biological and three technical repetitions

**Fig. 8:**
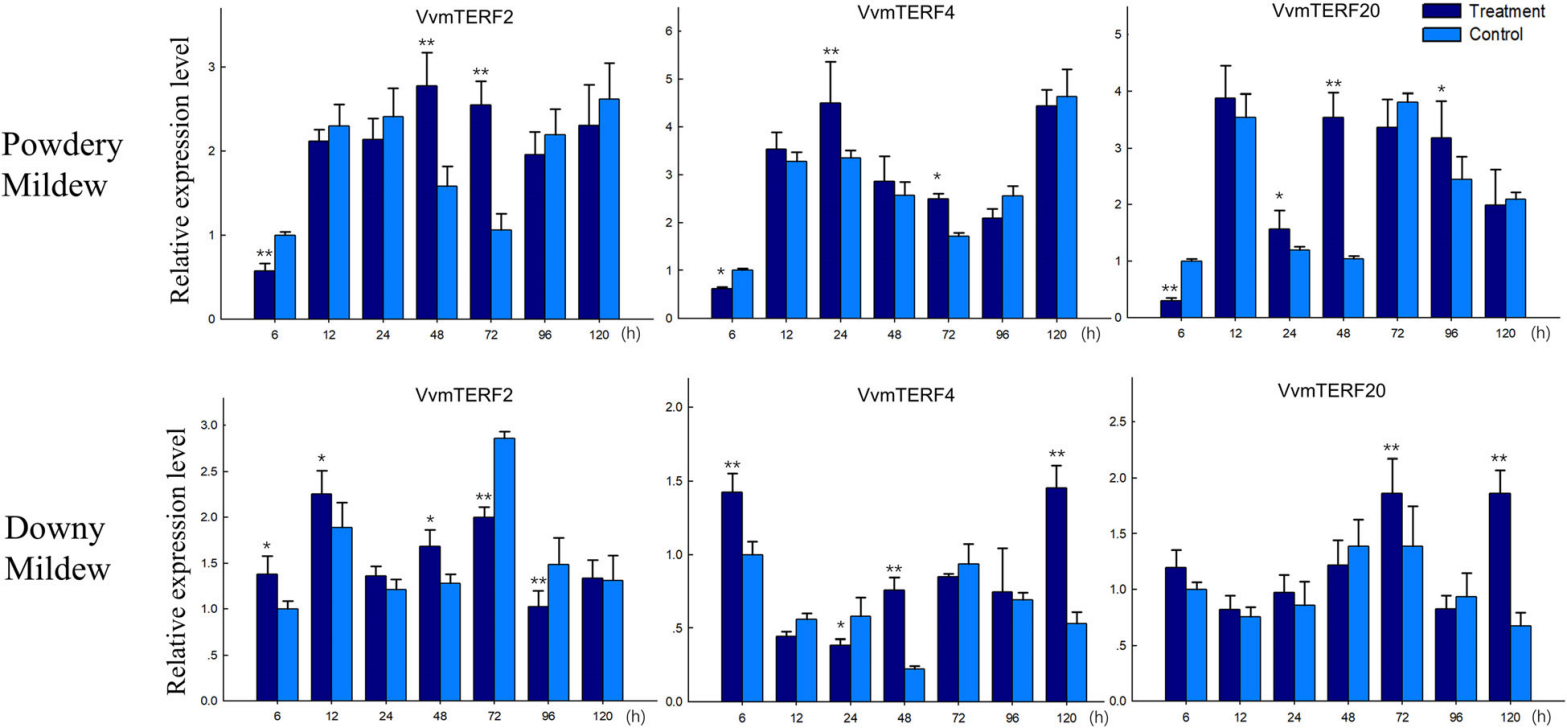
Real-time quantitative PCR expression patterns of 3 selected grape mTERF genes regulated by inoculating powdery mildew and downy mildew pathogens. Asterisks indicate that the corresponding genes were distinctly up- or down-regulated following different treatments by t-test (*P < 0.05, **P < 0.01). The mean and SD values were derived from three biological and three technical repetitions

In order to adapt to changing environments, the ability to tolerate diverse array of stresses is an essential evolutionary trait in plant kingdom. Identification and functional analysis of genes involved in biological signal transduction pathways is of great significance in providing a fundamental information for plant development and stress responses. To investigate their role in responding to biotic stress, express analysis of the 25 *VvmTERF* genes were conducted in potted ‘Thompson Seedless’ grapevines in greenhouse after inoculating with powdery mildew (PM) and downy mildew (DM) pathogens. As shown in Fig. 6, most *VvmTERF* genes demonstrated a tendency of downward expression after the inoculation. For instance, the expression of clade VII genes-*VvmTERF5*, *7*–*12*-decreased in both *E. necator* and *P. viticola* treatments, while *VvmTERF7* and *VvmTERF10* genes have slightly decreased after *P. viticola* inoculation (Fig. 6, Fig. S3 and S4). Besides, the expression level of *VvmTERF6, VvmTERF14* and *VvmTERF19* held steady under both biotic treatments. On the other hand, *VvmTERF11*, *VvmTERF17* and *VvmTERF21* displayed an increasing trend in both PM and DM treatments in comparison with the control. Based on semi quantitative RT-PCR analysis, three grape mTERF genes (*VvmTERF2*, *VvmTERF4* and *VvmTERF20*) were chosen for further detailed analysis using real-time qPCR (Figs. 7 and 8). The qPCR results were consistent with the those obtained by semi quantitative RT-PCR.

## Discussions

Widely identified in metazoans and plants, mitochondrial transcription termination factors (mTERFs) can regulate the expression of organelle genes at different levels [36, 37]. Previous research showed that mTERF plays a significant regulatory role in the evolution of mitochondrial genes, leading to a fundamental role in mitochondrial functionality, biological evolution, gene diagnosis and treatment [38]. In plants, the expression of mitochondrial genes are fundamental for various plant biological functions [39]. To fully explore the functions of grape mTERF genes, it is essential to identify and characterize mTERF genes in grape genome. In the current study, 25 grape mTERF genes were identified and their structures, evolutionary traits and expression patterns in responding to biotic stresses and hormone treatments were also analyzed.

### Identification, annotation and evolution of *VvmTERF* genes

In this study we investigated mTERF genes in grapevine and found 25 genes belonging to mTERF family that can be mapped onto the sequenced grape genome. The number of grape mTERF genes was less than that of Arabidopsis, despite the fact that grape has a much larger genome which would suggest the un-sequenced genomic gaps or mis-annotated genes of grape genome.

According to phylogenetic analysis, mTERF genes of Arabidopsis have been classified into eight groups [19]. In this study, a constructed phylogenetic tree which gathered the mTERF proteins from Arabidopsis, maize and grape had close topological framework to the tree constructed. Based on phylogenetic classification, grape mTERF genes were classified into nine groups. The number of clades I and VII of *VvmTERF* genes are small that could be due to a different pattern of duplication events. Furthermore, most of the *VvmTERF* genes were closely related to *AtmTERF* genes, which is in accordance with the fact that both grape and Arabidopsis are eudicots and exiting an appearance of close evolutional distance. As a result of highly conserved features, those mTERF genes which contain the same subclass may display similar functions. Multiple Arabidopsis mTERF genes functions have been tested, for instance, *AtmTERF5*, *9*, *10* and *11* have functions on the resistance to salt and osmotic stress, and *AtmTERF5*, *9* and *10* also play roles in responding to ABA regulation [40]. Although the Arabidopsis mTERF genes can provide predicted characterization for *VvmTERF* genes, the functional analysis of *VvmTERF* genes homologs still need more detailed experimental demonstration.

### Expansion and synteny analysis of *VvmTERF* gene family

During evolution, tandem, segmental and whole genome duplications have been commonly found in many organisms [34, 41]. In our study, based on the chromosome locations, motifs and sequences, we concluded that some of the *VvmTERF* genes such as *VvmTERF3–4* and *VvmTERF8–11* might arise by tandem duplications. This leads to rearrangement and extension of the mTERF gene family, reported in other plant species [20, 28, 42], such as Arabidopsis genome [43]. Some of the Arabidopsis mTERF genes were considered to be generated by tandem duplications and one block duplication [19, 20]. On the other hand, it has been demonstrated that grape has gone through whole-genome duplication events distinctly [44]. Therefore, tandem and segmental duplications could probably contribute for most gene extensions, although there are different opinions on the exact nature and timing of these events [44, 45]. Similarly, tandem and segmental duplications have probably played a key role for grape mTERF gene expansions and their structural and functional diversity. Therefore, according to the comparison with respective orthologs of the model plant Arabidopsis, the putative functions of grape mTERF genes can be speculated. The current work analyzed the tandem duplication events of the 25 grape mTERF genes on the 13 grape chromosomes based on the research techniques of Holub [46], within 200 kb length on all chromosomes containing more than two genes that will be deemed to regard as a tandem duplication event.

In order to research a less-studied species, we often use genomic comparison method which could effectively transfer genomic knowledge obtained from a well-studied model species (e.g. Arabidopsis) to a less studied organism [33, 47]. In this research, as seen in Fig. 3, synteny analysis of the grape and Arabidopsis genomes identified six pairs of *mTERF* genes (*VvmTERF2*-*AtmTERF6*, *VvmTERF6*-*AtmTERF4*, *VvmTERF13*-*AtmTERF19*, *VvmTERF15*-*AtmTERF10*, *VvmTERF24*-*AtmTERF9* and *VvmTERF25*-*AtmTERF17*) located in syntenic genomic regions (Fig. 3). Accompanied by selected genes loss, Arabidopsis and grape genomes have also gone through multiple and crucial chromosomal rearrangements and fusion processes during their evolution, which results in introduction of genes mismatches on chromosomes. In this case, we can deduce that the mTERF genes of grape and Arabidopsis in the same linear region may have a common ancestor. The first identified mTERF gene in Arabidopsis is *AtmTERF1* (*SOLDAT10*), which is mainly involved in fluorescent phenotype and O^2−^ signaling cell death [21]. Furthermore, the *AtmTERF4* (*BSM/RUG2*) gene is crucial for plant development. The *rug2–1* and *bsm* mutant are unable to grow compared with the wild type plant. Sequence analysis revealed that *VvmTERF6* was homologous to *AtmTERF4* which might imply that the *VvmTERF6* may have similar function in regulating plant development.

### *VvmTERF* genes response to hormone treatments and biotic stresses

In a previous study, Linder [10] firstly described the mTERF gene family in plants leading to number of studies on identification of this family in various plant species. Identification and functional analysis of mTERF gene family in maize and pepper are among the best examples [28, 42]. However, information about mTERF functions in plants is still rather limited and needs further investigations. In this report, we analyzed the expression patterns of *VvmTERF* after exposing to various biotic and abiotic stresses including pathogens and phytohormones. Under these different treatments, the *VvmTERF* genes showed various expression patterns, implying their crucial role in plant growth and response to environmental stresses.

Among the phytohormones, it has been reported that ABA is widely involved in various biological function in response to biotic and abiotic stress [48–50], while plant hormone Eth, SA and MeJA have synergistic effects on biological stress signals after pathogen infection [51]. Our results revealed that the grape mTERF genes responded to different plant hormones, which were consistent with former studies in other species such as maize and capsicum mTERF genes [28, 42].

A total of 35 mTERF genes were identified in *A. thaliana*, among which 6 mTERF genes were discovered specifically functional. Gene *SOLDAT10* and *SHOT1* for instance can respond to abiotic stresses [21, 52], as well as gene *TWIRT1* has meristem function [53] and gene *RUG2* is associated with leaf morphology [54]. *VvmTERF6* is a homolog of *AtmTERF4* that has functions of organelles development and photoautotrophic growth. In Fig. 5, expression alteration of *VvmTERF6* gene in the development stages of grape showed distinct upregulated pattern, indicating potential function during growth periods. It is interesting to note that 11 light responsive cis-elements were found from the promoter region of *VvmTERF6* gene in Fig. 4, suggesting a role in grape growth and development. Named *MDA1*, *AtmTERF5* played a role in rooting process of model plant Arabidopsis and showed responses to abiotic treatment. On account of reducing sensitivity to hormone abscisic acid, mutant *mda1* seedlings are exhibited insensitiveness to osmotic and salt stresses, while grown Arabidopsis *mda1* plants demonstrate reduced tolerance to cold, salinity or ABA treatment [22]. Hence, since this gene family has expanded in plants, scientists found that this family is a suitable candidate for many mutant-generating strategies in order to determine potent phenotypic, or even showed embryo-lethal feature. In addition to the results acquired from the mutant analysis, previously published mTERF genes expression data which had also clearly showed the potential role for mTERFs in plant stress response. In the report, *ZmmTERF12* and *ZmmTERF28* were down- and up-regulated by ABA hormone treatment, respectively [28]. From the same clade with *ZmmTERF28*, *VvmTERF25* also displayed quick response to ABA after 0.5 h treatment. It was found that abscisic acid responsive element appeared in the promoter region of *VvmTERF25*, implying the reason that ABA lead to a positive regulation of *VvmTERF25*. Taken together, we analyzed the response of *VvmTERF* genes to various plant hormones and found potential key targets for enhancing grape stress resistance, which provided basic information for future studies on function of *VvmTERF* genes and its related signal transduction network.

Previous research demonstrated that some of the plant mTERF genes such as *SOLDAT10* and *SHOT1* participate in ROS scavenging process [52]. Given this, the role of mTERF genes in response to biotic stresses needs more information. Further we conducted the related experiment here and the results confirmed our hypothesis before. In this study, we found that some grape mTERF genes responded to powdery or downy mildew treatments. For example, *VvmTERF22* was up regulated by PM at 12 h, and *VvmTERF21* positively responded to downy mildew infection. We detected several *VvmTERF* genes expression levels which are actively responsive to PM or DM treatments, indicating these genes might display significant characters in the grape protection, but further research is needful to demonstrate that they participate in biotic stress responses in grapevine.

## Conclusions

The family of transcriptional activators encoded by the mTERF genes is widely found in plants and animals. Although significant progresses have been made in identifying mTERF genes in model plant species, little information of mTERF genes has been known in fruit crops. In this study, we identified a total of 25 *VvmTERF* genes in the grape genome, and also investigated their structural, phylogenetical and syntenic features. Through comparative analysis of homology between grape and Arabidopsis, it is found that several mTERF genes of grape and Arabidopsis are located in the homologous regions, indicating that they may present close evolutionary relationship. Expression analysis of *VvmTERF* genes showed that multiple genes could respond to different biological stresses and hormone treatments. Results from this study have paved the way for future research to investigate roles of *VvmTERF* genes on disease resistance of grapevines.

## Methods

### Identification and annotation of grape mTERF genes

Conserved mTERF domains were first used to detect grape genes in mTERF HMM (Hidden Markov Model) file (PF02536) from the Pfam database [29] using the HMMER 3.0 package [26]. The domains were further used as the query to search the GenBank nonredundant protein and the Grape Genome Database (http://www.rosaceae.org/projects/grape_genome) using the BLAST program. All mTERF proteins with an E value < 0.01 were collected and the domains were manually checked in each candidate *VvmTERF* gene.

### Multiple sequence alignment, phylogenetic analysis and classification

A total of 25 *VvmTERF* genes containing mTERF core domains were identified. The CLUSTALX software was then used for multiple sequence alignment analysis including grape mTERF genes and those from Arabidopsis (*AtmTERF*) and maize (*ZmmTERF*) [32]. Based on the neighbor-joining method and maximum likelihood method for 1000 bootstrap replicates, a phylogenetic tree was constructed using MEGA5.0 software [55]. The *VvmTERF* genes were classified into clades ground on multiple sequence alignments with those *AtmTERF* and *ZmmTERF* genes.

### MEME motifs, conserved sequences and subcellular localization analysis of grape mTERF proteins

The identification of known conserved motifs in grape mTERF proteins was conducted by BLAST against the SMART [30] and Pfam [29] database searching. The potential motifs in the putative mTERF family gene sequences were predicted by Multiple Em for Motif Elicitation (MEME) software [31] with the parameters as follow: the optimum width of every single motif distributed between 6 to 50, and the maximum number of motifs to find was 15. After that, the collection and cluster of mTERF motifs from grape mTERF proteins were conducted using the ClustalW 2.0 program software [32], and graphical representation of amino acid residues was arranged by TBtools [56].

The presumptive subcellular localizations of VvmTERF proteins were predicted by VvmTERF protein sequences in the online program Cellov2.5 Server (http://cello.life.nctu.edu.tw/) [57].

### Exon–intron structure analysis, synteny analysis and gene duplication

According to alignments of grape mTERF gene coding sequences and their respective full-length sequences, the exon-intron structure was determined on Grape Genome Browser: http://www.genoscope.cns.fr/externe/GenomeBrowser/*Vitis*/. And the online program Gene Structure Display Server (GSDS: http://gsds.cbi.pku.edu.ch) [58] was carried out to obtain relative diagrams. Besides, the definition of mTERF genes with tandem duplication events were adjacent homologous genes on a single chromosome, while gene duplication events among diverse chromosomes were defined as segmental duplications [59]. The specific physical location of each *VvmTERF* gene on its individual chromosome determines whether it is considered in a tandem duplication event. Therefore a synteny analysis map of grape mTERF gene was constructed via the syntenic blocks, and a further synteny analysis between grape and *AtmTERF* genes was acquired from the Plant Genome Duplication Database [60]. The generation of related diagrams were illustrated using Circos version website (http://circos.ca/).

### Cis-element analysis of grape *mTERF* genes promoter

The 2000 bps upstream promoter sequence of each grape mTERF gene coding regions were obtained from the grape genome database (https://wwwdev.genoscope.cns.fr/vitis). PlantCARE online analysis program (http://bioinformatics.psb.ugent.be/webtools/plantcare/html/) was used to find the predicted cis-element.

### Expression profiles of grape *mTERF* gene family in different tissues and organs

The expression profile of grape mTERF gene family was confirmed in a *V. vinifera* cv ‘Corvina’ (clone48) gene expression atlas of various organs at different developmental stages. Microarray data were collected from the NCBI gene expression omnibus (GEO) datasets under the series entry GSE36128 (http://www.ncbi.nlm.nih.gov/geo/) [61]. The mean expression value of grape mTERF genes in all tissues and organs were analyzed and detailed displayed by Multiple Experiment Viewer software (MeV) [62]. Measured using RNA-Seq data, the expression patterns of *VvmTERF* genes in various berry developmental stages were gained from gene expression omnibus (GEO) database of NCBI (GSE77218) [63].

### Plant materials and stress treatments

To validate the expression regulation of grape mTERF genes under abiotic and biotic stresses, grape leaves and organs were obtained from *V. vinifera* ‘Thompson Seedless’ grape grown in a greenhouse. When the shoots of the grapevines reached 30 cm long with fully expanded young leaves, the plants were subjected to hormone treatment. Hormone treatment was carried out on grape leaves under similar growth condition, spraying with 100 μM salicylic acid (SA), 300 μM abscisic acid (ABA), 50 μM methyl jasmonate (MeJA), and 0.5 g/l ethylene (Eth), respectively [34]. Leaves from the treated vines were collected at 0.5, 1, 3, 6, 12, 24, and 48 h post treatment. Grape leaves sprayed with sterile water were used as the negative control.

In term of biotic stress, powdery mildew (*Erysiphe necator*) and downy mildew (*Plasmopara viticola*) pathogens were used to inoculate young leaves of *V. vinifera* ‘Thompson Seedless’ following previous protocol [64, 65]. Leaves were sampled at 6, 12, 24, 48, 72, 96, and 120 h post inoculation and untreated leaves were collected as the negative control. At each time point of all treatments, nine leaves from three separate plants were homogenized, and the treatments were conducted three times independently. These grape leaves were frozen in liquid nitrogen and stored at − 80 °C for further use.

### Semiquantitative PCR and real-time quantitative PCR analysis

The total RNA was extracted as described by Zhang et al. [47]. The genomic DNA was digested using RNase-free DNase I kit (OMEGA Bio Inc., USA). The grape *Actin1* gene (GenBank Accession number AY680701) and *EF1-α* (GenBank Accession number EC931777) gene were chosen as housekeeping genes and amplified with the primers showing in Table S1, which also includes Gene-specific primers for the 25 *VvmTERF* genes. For the semi-quantitative reverse transcription-PCR experiment, the volume of reaction system was 20 μL which includes 1 μL cDNA template, 1 μL forward and reverse gene-specific primers (10 μM), 10 μL PCR Master Mix (Qingke Biotech Co. Ltd., Shanghai, China) and 8 μL sterile water, the specific proportion and program were set according to the PCR Master Mix instruction book. Each PCR reaction was conducted in duplicate. The Gene Tools software was used for quality control of semi-quantitative PCR results, further log-transformed values of the relative expression patterns of *VvmTERF* genes under various phytohormone and biotic stresses treatment were used to perform hierarchical cluster using Mev software [62].

Quantitative real-time PCR analysis was performed with an IQ5 real-time PCR instrument (Bio-Rad, Hercules, CA, USA). All reactions were performed in triplicate with a reaction system of 20 μL including 1 μL specific primers (10 μM), 1 μL cDNA, 10 μL SYBR green (Yeasen Biotech Co Ltd., Shanghai, China), and 8 μL sterile water, the specific proportion was on the instruction book as well as the PCR parameters. The grape Actin1 gene (GenBank Accession number AY680701) was chosen as the housekeeping gene. The expression levels of grape mTERF genes were analyzed using IQ5 software with the normalized expression method. The t-test was conducted using the SPSS software (SPSS 17.0, Chicago, IL, USA, *P < 0.05, **P < 0.01).

## Supplementary Information

**Additional file 1: Figure S1.** Phylogenetic analysis among the grape, Arabidopsis and maize mTERF proteins. The unrooted tree was constructed using MEGA5.0 software by Maximum Likelihood method for 1000 bootstrap replicates. Three dot colors mean different species. Yellow, green and red represent maize, Arabidopsis and grape, respectively.

**Additional file 2: Figure S2.** Expression patterns of 25 *VvmTERF* genes under hormone Eth, MeJA, SA and ABA treatments analyzed by semi-quantitative RT-PCR. *Actin1* and *EF-1α* (GenBank Accession number AY680701 and EC931777) were used as internal reference genes. The upper and lower bands indicate treatment and control, respectively.

**Additional file 3: Figure S3.** Expression patterns of 25 *VvmTERF* genes after inoculation of downy mildew and powdery mildew treatments analyzed by semi-quantitative RT-PCR. *Actin1* and *EF-1α* (GenBank Accession number AY680701 and EC931777) were used as internal reference genes. The upper and lower bands indicate treatment and control, respectively.

**Additional file 4: Figure S4.** Expression profiles of 25 *VvmTERF* genes under exogenous hormone and biotic treatments. Numbers in boxes represent different expression levels. 0 indicates no change, number less than zero means down-regulated expression and more than zero means up-regulated expression.

**Additional file 5: Table S1.** Primer sequences used for semi-quantitative RT-PCR and quantitative real-time PCR of the 25 grape mTERF genes. Primer 5.0 software were used to designed specific primers.

**Additional file 6: Table S2.** Synteny blocks of *VvmTERF* genes between the grape and Arabidopsis genomes.

## Data Availability

All data generated or analyzed during this study are included in this published article and its additional files.

## Abbreviations

mTERFs: Mitochondrial transcription termination factor
VvmTERFs: *Vitis vinifera* mitochondrial transcription termination factor
PPRs: Pentatricopeptide repeat proteins
HAT: Half atetratricopeptide proteins
OPR: Octotricopeptide repeat proteins
rRNA: Ribosomal RNA
Eth: Ethylene
SA: Salicylic acid
MeJA: Methyl Jasmonate
ABA: Abscisic acid
RT-qPCR: Reverse transcription quantitative PCR
