# Supplementary figures and images for: Identification, characterization and functional analysis of grape (Vitis vinifera L.) mitochondrial transcription termination factor (mTERF) genes in responding to biotic stress and exogenous phytohormone
Source: BMC Genomics. 2021 Feb 26;22:136. doi: 10.1186/s12864-021-07446-z (PMC7913399; doi:10.1186/s12864-021-07446-z)

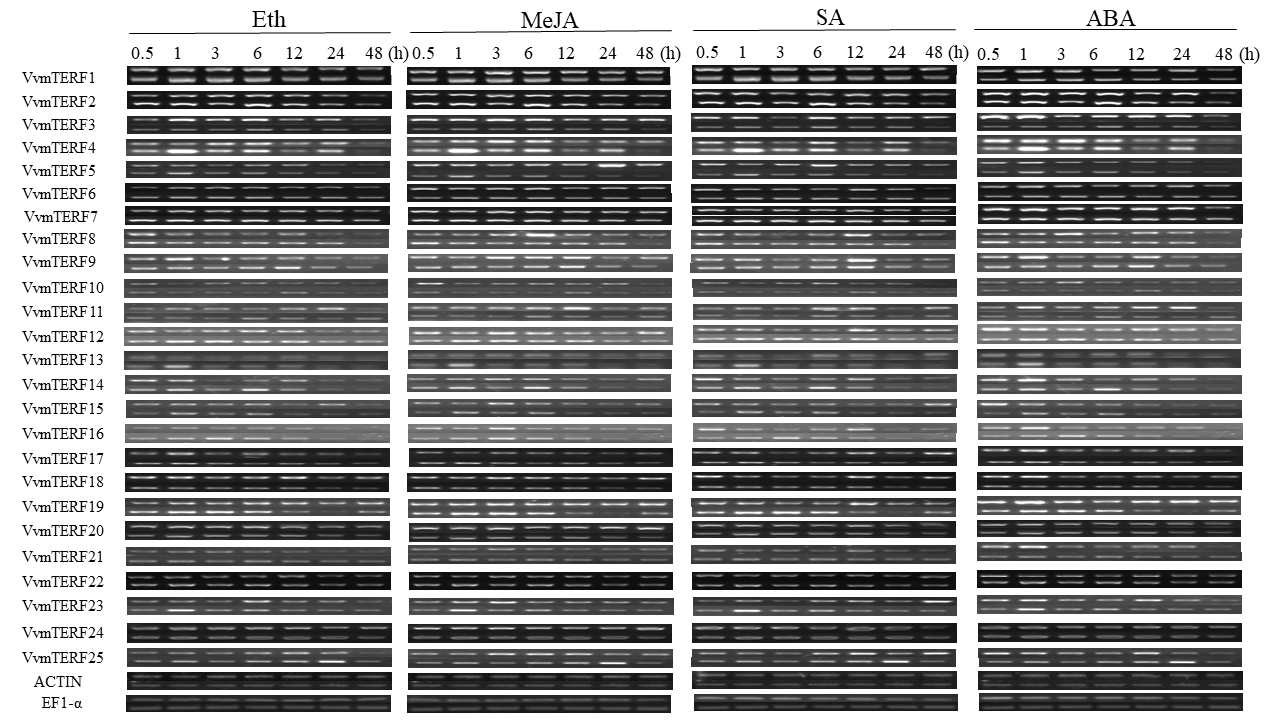

Supplement: Supplementary file 2 — Additional file 2: Figure S2. Expression patterns of 25 VvmTERF genes under hormone Eth, MeJA, SA and ABA treatments analyzed by semi-quantitative RT-PCR. Actin1 and EF-1α (GenBank Accession number AY680701 and EC931777) were used as internal reference genes. The upper and lower bands indicate treatment and control, respectively. [file 12864_2021_7446_MOESM2_ESM.tif]

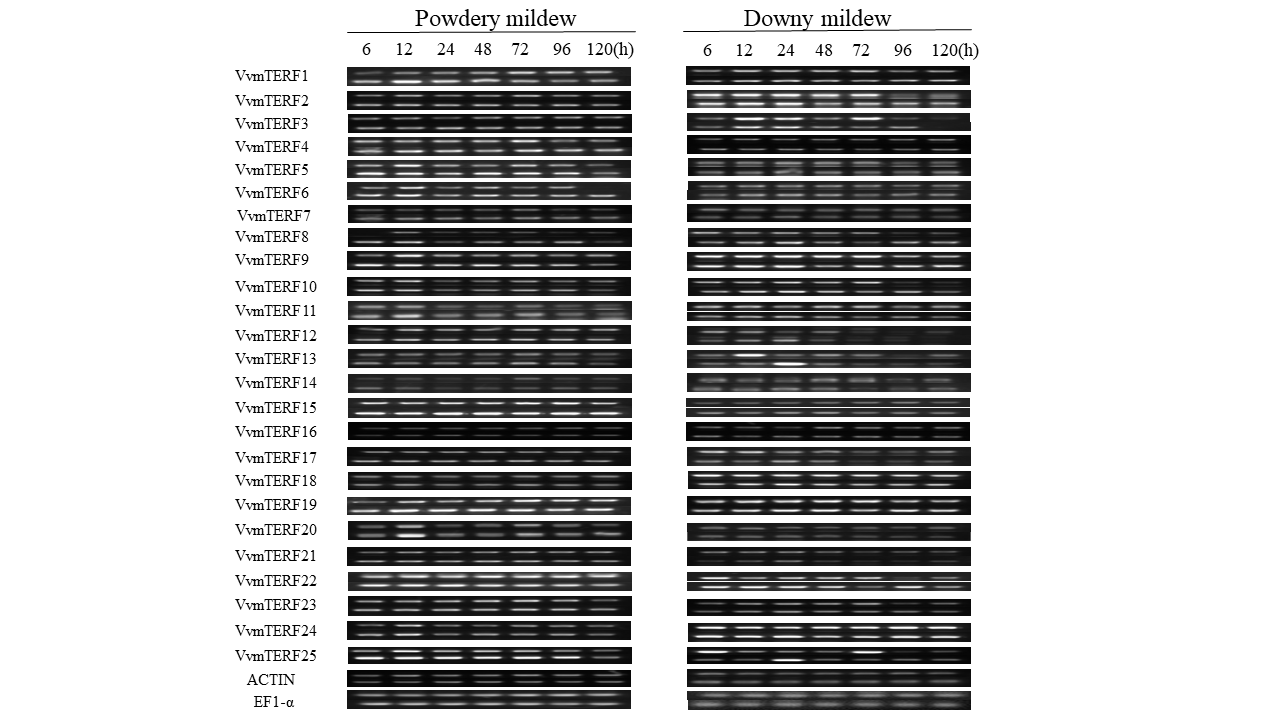

Supplement: Supplementary file 3 — Additional file 3: Figure S3. Expression patterns of 25 VvmTERF genes after inoculation of downy mildew and powdery mildew treatments analyzed by semi-quantitative RT-PCR. Actin1 and EF-1α (GenBank Accession number AY680701 and EC931777) were used as internal reference genes. The upper and lower bands indicate treatment and control, respectively. [file 12864_2021_7446_MOESM3_ESM.tif]

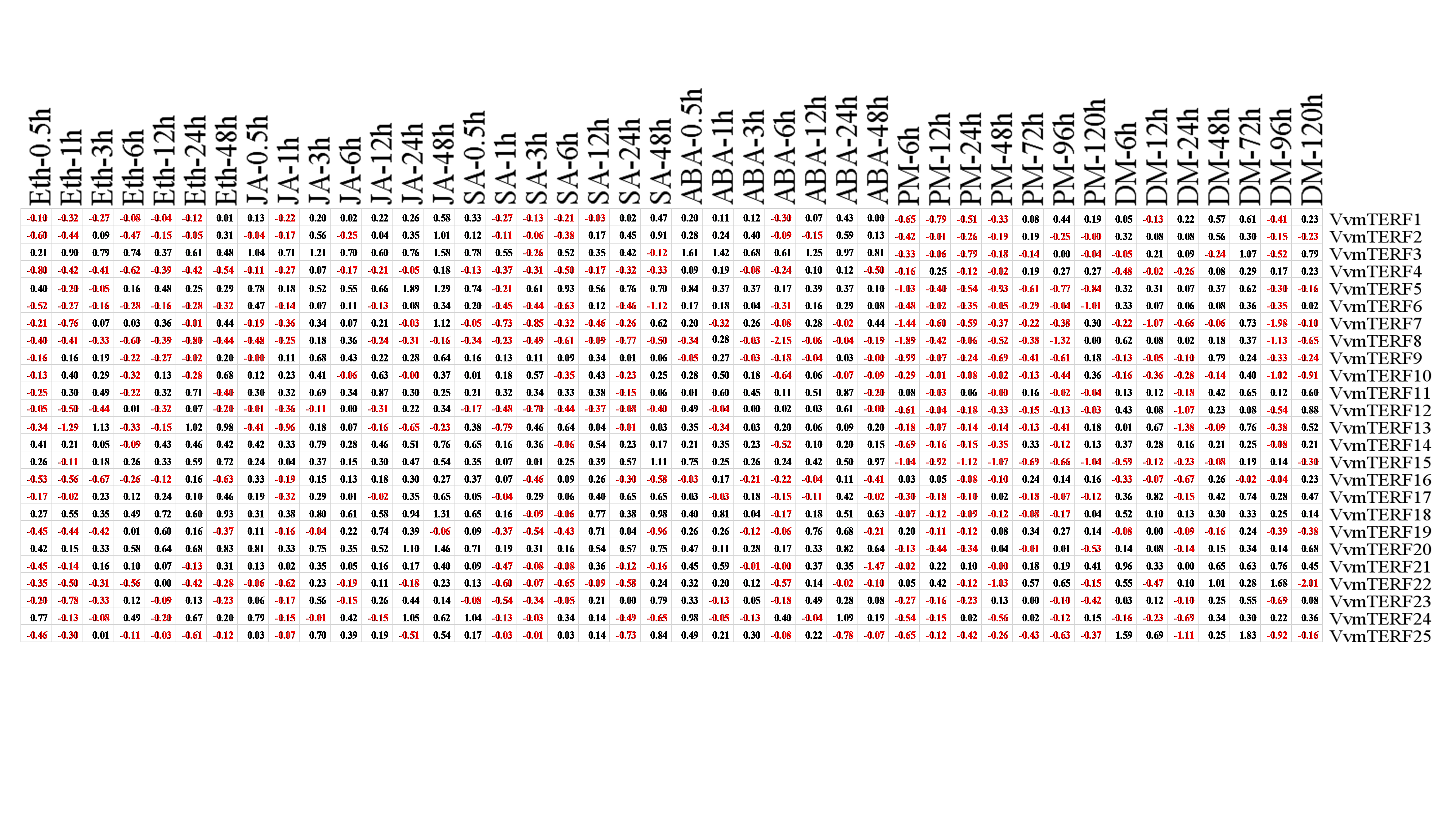

Supplement: Supplementary file 4 — Additional file 4: Figure S4. Expression profiles of 25 VvmTERF genes under exogenous hormone and biotic treatments. Numbers in boxes represent different expression levels. 0 indicates no change, number less than zero means down-regulated expression and more than zero means up-regulated expression. [file 12864_2021_7446_MOESM4_ESM.tif]
